# Scalable deep text comprehension for Cancer surveillance on high-performance computing

**DOI:** 10.1186/s12859-018-2511-9

**Published:** 2018-12-21

**Authors:** John X. Qiu, Hong-Jun Yoon, Kshitij Srivastava, Thomas P. Watson, J. Blair Christian, Arvind Ramanathan, Xiao C. Wu, Paul A. Fearn, Georgia D. Tourassi

**Affiliations:** 1 Biomedical Sciences, Engineering, and Computing Group, Health Data Science Institute, Oak Ridge National Laboratory, Oak Ridge, TN USA; 2 Herff College of Engineering, University of Memphis, Memphis, TN USA; 3 Louisiana Tumor Registry, Louisiana State University Health Sciences Center, New Orleans, LA USA; 4 Surveillance Research Program, National Cancer Institute, Bethesda, MD USA

## Abstract

**Background:**

Deep Learning (DL) has advanced the state-of-the-art capabilities in bioinformatics applications which has resulted in trends of increasingly sophisticated and computationally demanding models trained by larger and larger data sets. This vastly increased computational demand challenges the feasibility of conducting cutting-edge research. One solution is to distribute the vast computational workload across multiple computing cluster nodes with data parallelism algorithms. In this study, we used a High-Performance Computing environment and implemented the Downpour Stochastic Gradient Descent algorithm for data parallelism to train a Convolutional Neural Network (CNN) for the natural language processing task of information extraction from a massive dataset of cancer pathology reports. We evaluated the scalability improvements using data parallelism training and the Titan supercomputer at Oak Ridge Leadership Computing Facility. To evaluate scalability, we used different numbers of worker nodes and performed a set of experiments comparing the effects of different training batch sizes and optimizer functions.

**Results:**

We found that Adadelta would consistently converge at a lower validation loss, though requiring over twice as many training epochs as the fastest converging optimizer, RMSProp. The Adam optimizer consistently achieved a close 2nd place minimum validation loss significantly faster; using a batch size of 16 and 32 allowed the network to converge in only 4.5 training epochs.

**Conclusions:**

We demonstrated that the networked training process is scalable across multiple compute nodes communicating with message passing interface while achieving higher classification accuracy compared to a traditional machine learning algorithm.

## Background

### Introduction

Deep Learning (DL) has recently advanced the state-of-the-art in bioinformatics applications ranging from medical imaging, drug discovery, to genomic medicine [1]. Deep learning models or Deep Neural Networks (DNNs) are characterized by their ability to automatically discover and abstract highly effective latent features from a wide variety of data [2]. Though deep learning alleviates traditional statistical machine learning’s need for manual, task-specific feature engineering, DL models can have thousands, sometimes a million or more times more numerical parameters to optimize during model training. Though the accumulation of massive training datasets has made training DL models possible, it compounds the practical issue of required computational resources. Although the computational challenge was initially addressed by utilizing specialized Graphics Processing Units (GPUs) [3], the exponential growth of dataset sizes enabled the use of these DL models in many domains.

One such domain to benefit from these advances in modeling is cancer surveillance – the timely, systematic collection and analysis of information relating to new cancer cases, extent of disease, treatment, survival, and cancer deaths [4]. In cancer surveillance, the scale of the data comes from the number of individuals affected. Over 1,685,000 new cases were diagnosed and there were over 595,000 deaths in 2016 in the United States alone [5]. Massive datasets aggregated over the course of each individual’s cancer diagnosis, treatment, and outcomes can help identify long-term trends and underlying patterns across widespread populations of people and ultimately demonstrate effectiveness of treatments and other control measures. The automation of information extraction and other tasks with advanced DL methods require new, scalable approaches to successfully acquire insights from these growing datasets in a feasible amount of time.

One solution to increase performance is to increase the computational resources with additional machines. Previous researchers have observed the effectiveness of running the stochastic gradient descent (SGD) optimization algorithm on multiple computational units reading and updating asynchronously from parameter weights in a shared memory configuration [6]. This approach’s theoretical basis was shown by Tsitsiklis et al. who demonstrated that asynchronous SGD optimization would converge if the delay between parameter updates and reads are bounded [7]. Subsequent researchers applied asynchronous training on the multi-layer perceptron DL model in a distributed memory computing environment by distributing data subsets to worker cluster nodes, known as data parallelism [8]. Recently more advanced alternatives to SGD were shown to improve performance rapidly by decreasing the number of optimization steps required for model convergence [9–11], though usage in a distributed environment remain underexplored.

In this paper, we applied data parallelism for the distributed training of an advanced deep learning model with large-scale datasets using a High-Performance Computing (HPC) environment. We implemented the distributed training of a DL model for the natural language processing (NLP) task of information extraction from a cancer pathology dataset too large to train feasibly on a single machine. We evaluated algorithm scalability by comparing time and performance variation across different optimization schemes, the number of compute nodes, and other training options.

### Cancer pathology reports and information extraction

Cancer pathology reports are a primary source of information for the Surveillance, Epidemiology, and End Results (SEER) program. The SEER program is the premier population cancer surveillance program covering approximately 28% of the United States (US) population [12]. Is the US, SEER is an important national resource for monitoring cancer outcomes across demographic groups, geographic regions, and over time. Furthermore, SEER provides unique insights into the impact of oncology practice outside the clinical trial setting. The information collected includes data on patient demographics, primary tumor site, tumor morphology and stage at diagnosis, the first course of treatment, follow-up for vital status, and more. Presently this data is mainly extracted by human coders, and a machine learning approach to automate the task would enable not only faster extraction of quality data, but also enable new fields to be extracted from historical data.

One such opportunity for automating information extraction from text comes from pathology reports. Pathology reports are unstructured text documents containing detailed descriptions of human tissue specimens. They are a standard component of clinical reporting and management of cancer patients. Much of this patient data is encoded in plain text pathology reports, and manual human annotations are labor intensive, costly, and error prone. Therefore, automatic information extraction from natural language text, which is an essential application in big data science, has received much attention from the cancer surveillance research communities.

In the past few years, text information extraction approaches to have advanced from strictly defined, manually tuned, rule-based classification algorithms [13] to statistical machine learning techniques like Support Vector Machines, Logistic Regression models, and Random Forest classifiers [14]. These previous approaches used the vector space model which represents documents as sparse high dimensional vectors with each dimension corresponding to a specific word and the value corresponding to that word’s occurrence or prevalence within the document [15].

Recently, DL methods have improved upon this framework in two major ways. First, word embeddings allow documents to be represented as a sequence of trainable dense word vectors preserving a document’s word ordering as in [16]. Secondly, Convolutional Neural Networks (CNNs) [2], originating in Machine Vision, have been applied recently to natural language understanding, achieving superior performance [17]. CNNs for NLP train many convolutional filters to quantify the relative usefulness of every word vector sequence in a document, then use Max-Pooling down sampling to select and feed forward only the most effective word features. Although the CNN eliminates the need for vector space models’ manual feature engineering, they have vastly more parameters which require more computationally demanding training.

Scaling deep networks like the CNN for NLP tasks is particularly critical for applications such as the one outlined above for cancer surveillance. These networks have the capability to identify complex structures and relationships between word features and labels that might be too complex for manual effort. It would not be feasible to implement these models without approaches to scale them beyond single-node computers or small-scale cluster computing environments. This paper investigates the feasibility of large-scale CNN training using data parallelism in an HPC environment with a large natural language text dataset of cancer pathology reports. The approach presented in this study is applicable to existing supercomputers as well as upcoming exascale computers.

### Data parallelism and distributed optimization

Data parallelism is a method for distributing a computational task across multiple compute/worker nodes by partitioning a training dataset [18]. In the DL context, this approach is initialized by the parameter server, which first partitions the training data then distributes the subsets to each worker node which subsequently commences local training. Each worker samples without replacement a portion or batch, of the training data for local network inputs to evaluate a loss function, then applies the back-propagation algorithms to obtain network weight gradients to submit to the master or parameter server. The parameter server accepts the workers’ gradients scaled by an optimizer function and returns updated parameter estimates to the workers. One can design the parallel training algorithm to wait for each worker node to contribute their gradient calculation update steps before returning updated parameter estimates back to the workers [8]. This “synchronous” approach is adequate if all the computing nodes possess the same computational power and have very similar gradient computation times. This type of algorithm is simple to implement in a cluster-computing environment, but if one node fails or is delayed for any reason, the entire training procedure is interrupted losing valuable compute time. In contrast, the “asynchronous” approach updates weights independently of the other compute nodes. This prevents the slower compute nodes from wasting training time on faster nodes, though a resulting drawback is that the lack of coordination may result in slower compute nodes submitting gradients with outdated network weight parameters.

## Methods

### Cancer pathology report dataset

The study was designed with electronic pathology reports provided by the Louisiana Tumor Registry using an IRB approved protocol. The dataset consists of 256,816 pathology documents, of which approximately 10% have human-annotation available for primary cancer site code as defined by International Classification of Diseases for Oncology, Third Edition (ICD-O-3) [19]. For our cross validated tests, we used site codes with at least 10 documents available, which resulted in 22,976 manually annotated documents (cases) with 64 primary cancer sites represented. ICD-O-3 codes and the corresponding number of cases associated with each code are listed in Table 1. We then divided the data into 10 folds balanced by class in preparation for 10-fold cross-validation experiments.

**Table 1:** Primary cancer site codes and corresponding number of pathology reports associated with the code

Code	# cases	Code	# cases	Code	# cases	Code	# cases
C00	16	C17	156	C41	88	C64	458
C01	53	C18	1951	C42	1800	C65	20
C02	108	C19	118	C44	1151	C66	32
C04	39	C20	646	C48	84	C67	947
C05	31	C21	75	C49	261	C68	30
C06	48	C22	268	C50	4415	C69	18
C07	66	C23	27	C51	97	C70	17
C08	16	C24	26	C52	39	C71	296
C09	71	C25	151	C53	314	C72	36
C10	27	C26	32	C54	882	C73	305
C11	43	C30	47	C55	174	C74	10
C12	11	C31	15	C56	448	C75	22
C13	14	C32	240	C57	83	C76	196
C14	24	C34	1570	C60	18	C77	741
C15	199	C38	106	C61	2313	C80	962
C16	427	C40	14	C62	60	C90	24

To process the pathology report text, we first split each document into words by separating the document’s character string on white space character tokens. We then removed all punctuation and set all alphabetic characters to lowercase. Tokens with a frequency of at least five were mapped to a unique index number, with all others mapped to a token corresponding to rare words.

### Convolutional neural networks for natural language processing

Unlike conventional machine learning approaches, the CNN for natural language processing [17] uses learned latent representations of words for document representation known as word vectors. To prepare pathology report text for classification, we tokenized each document on the word and non-alphanumeric symbol and set all alphabetical characters to lowercase. We substituted all decimal numbers to a single “float” token and all 3 or more digit integers to a “large integer” token. We then identified all tokens with a document frequency of less than five and mapped them to a token to represent rare tokens. The tokens with a document frequency of five or more comprised our vocabulary. For each token in our vocabulary along with the rare tokens, we randomly initialized a word vector of length 300 from a uniform distribution with bounds (−.025, .025). This dimensionality is common for similar information extraction tasks [16], while also empirically shown to be effective for this particular network [20]. With this preprocessing, we found that over 98% of reports had a token length of less than 1500, therefore we truncated the longer reports to 1500 tokens and we padded the shorter reports with a zero vector. As a result, each pathology report was represented by a 1500 × 300 matrix.

In the context of deep neural networks, a convolution is an automatic feature generation technique which processes an input with a learnable regional filter spanning a specified length of words. We can apply a convolutional filter to the document matrix with a linear filter with region size *h* that corresponds to a context length of *h* word vectors. Specifically, we can parameterize a linear filter as a weight matrix *w* with dimensions (*h* × *k*). A context window for matrix *A* starting from the *i*-th word vector of *A* with length *h* can be represented by submatrix *A*[*i* : *i* + *h* − 1] [20]. A single convolution on the document’s *i*-th word with context length *h* can be denoted as *o*_*i*_ = *w* · *A*[*i* : *i* + *h* − 1] where *o*_*i*_ ∈ *ℝ*^(*n* − *h*)^. Finally, we apply an activation function *f* with a bias term *b* ∈ *ℝ* to *o*_*i*_, inducing a single feature map *c*_*i*_ = *f*(*a*_*i*_ + *b*). The final output of a convolutional filter over document matrix *A* can be expressed as feature mapping *c* = {*c*_1_, *c*_2_, …, *c*_*n*_}, which is a representation of each context of *h* words over the document matrix *A* [17]. Global max pooling then down samples the feature mapping to a single scalar, which is concatenated with the other down-sampled feature mappings and fed through a penultimate fully-connected hidden layer and finally a softmax classification layer.

Through the feature mapping mechanism, the CNN attempts to extract representations of word sequences and then selects the most relevant context when classifying a pathology report, producing a “feature mapping” to represent each context size. The subsequent pooling layer trains the convolutional filter to extract a single feature map scalar from each mapping, aggregating the selected contexts by concatenation. This pooling is a highly efficient form of feature selection which allows our model to classify documents by learning to identify the most important word context [21].

In all our experiments, we used convolutional filters with 300 filters for token lengths of 3,4, and 5. For model regularization, we applied dropout at a rate of .5 on the hidden layer and applied *l*_2_-normalization on our weight vectors. We also applied *l*_2_-normalization on the word vector matrix, which we found substantially improved accuracy for the under represented classes.

### Data parallelism on high-performance computing

In this study, we selected the downpour Stochastic Gradient Descent (SGD) as our distributed training algorithm [8]. The algorithm is initialized as follows: Let *N* be the number of compute nodes available, and *M* be the total number of training cases. The train set is partitioned into *N* training sets with approximately *M*/*N* training cases each. The training sets are then scattered so that each compute node *n* manages its own local network weights ${w}_n^t$. Furthermore, with the asynchronous distributed approach, we have an additional master parameter process which aggregates our compute node updates, thereby requiring a total of *N* + 1 nodes for our asynchronous data parallelism implementation.

Our distributed training algorithm is as follows: a mini-batch of labeled training data on compute node *n* is fed through the model with weights ${w}_n^t$ to calculate cross entropy loss and resulting gradients $\Delta {w}_n^t$. These updates are sent to the parameter server. The parameter server’s weights *w*^*t*^ are updated using the received compute node gradients with a pre-specified optimization rule or optimizer denoted *opt*(·), such that the updated parameter server weights are updated as ${w}^{\left(t+1\right)}={w}^t- opt\left(\Delta {w}_n^t,\theta \right)$, where *θ* is the set of optimizer parameters, such as the learning rate. Finally, the updated parameter server weights *w*^(*t* + 1)^ are distributed to each compute node. If compute nodes receive parameters during mini-batch computations, the master node weight updates are locally applied only after the mini batch’s gradients are calculated and sent to the parameter server. This means parameter server updates might not necessarily have been computed with the parameter server’s latest weights. Because the worker nodes do not compute additional mini-batches until current gradients have been accepted and updated by the parameter server, this lag is bounded.

Before running our scalability experiments, we must select an optimizer. We compared the relative effectiveness of four popular optimizers: Stochastic Gradient Descent (SGD), Root Mean Square Propagation (RMSProp), modified adaptive gradient (Adadelta), and adaptive moment estimation (Adam).

SGD is the most basic optimizer and updates the parameter server weights simply by subtracting mini batch gradients scaled by a pre-defined static learn rate [22]. RMSProp optimizer implements an “adaptive learn rate” by dividing SGD’s learn rate by the exponentially decaying average of squared gradients, gradually reducing the learn rate as the gradients decrease [9]. Adadelta attempts to improve adaptive learning by removing the learn rate parameter for weighted average of current and past gradients within a specified number of iterations [10]. Lastly, Adam iterates on RMSProp by additionally scaling the learn rate with exponentially decaying average of past gradients in addition to RMSProp’s exponentially decaying average of past gradients squared, which allows for adaptive learn rates similar to RMSProp but with smoother update values [11].

### Experiment design

For our experiments we utilized the implementation of downpour SGD algorithm [23] using the Keras DL library [24] with a TensorFlow [25] backend on the Titan supercomputer at the Oak Ridge Leadership Computing Facility (OLCF), Cray XK7 architecture with 18,688 nodes. Each node has an NVIDIA Tesla K20 GPU with 6GB graphics memory. Gradients and updated weights are transferred with Message Passing Interface (MPI).

We performed three information extraction experiments using the CNN model to predict the primary ICD-O-3 cancer site. For our experiments, we split our data into 10-fold class-stratified cross validation folds using one fold as a test set and the remaining folds as our training set. Furthermore, we randomly partitioned 25% of our training data to use as a validation set.

In our first two experiments exploring the optimal choice of optimizer, number of worker nodes, and mini-batch size, we trained our network on only one fold and reported the cross-entropy loss evaluated on the validation set.

We first attempted to compare the performance between our four optimization algorithms by measuring how quickly the algorithm converges to a minimum cross-entropy loss and the lowest minimum loss convergence value. For this first experiment we used 16 worker nodes and compared the Adadelta, Adam, RMSProp, and SGD optimization algorithms using training mini-batch sizes of 16, 32, 64, 128, and 256. We trained our CNN using each optimizer-batch size configuration for 25 epochs, and recorded validation loss after training on 1/4 of each epoch.

To explore scalability of our distributed learning algorithm, we compared how utilizing additional worker nodes with different batch sizes impacts the elapsed training time and minimum validation loss achieved using a particular optimizer. Using either 4, 8, 16, or 32 worker nodes training with mini-batches of size 16, 32, 64, 128, or 256, we trained our CNN for 10 epochs while obtaining validation metrics in a manner identical to the previous experiment.

Lastly, we evaluated the performance of the CNN with results from all 10-fold cross validation experiments with the choice of the best optimizer, number of worker nodes, and mini-batch size determined by the first two experiments. Each fold of the CNN was trained using 16 worker nodes and a batch size of 64 with the Adadelta optimizer, as we found this configuration achieved the lowest overall validation loss from our initial experiment. As a benchmark for comparison, we performed the same 10-fold cross validated experiments using the statistical learning Random Forest classifier, a commonly used supervised learning algorithm based on ensembles of decision trees. For the Random Forest classifier, we used the vector-space model document representation of Term Frequency / Inverse Document Frequency (TF-IDF) of 2000 *n*-grams (*n* = 1,2,3). We adopted micro and macro-averaging of F1 scores as our performance metrics, which are accepted in natural language processing community [26]. Micro-averaging gives equal weight to each classification decision, whereas macro-averaging gives equal weight to each class. These two metrics combined give a better understanding of how well the classifier behaves over class-imbalanced data.

## Results

### Optimizer choice

**Fig. 1:**
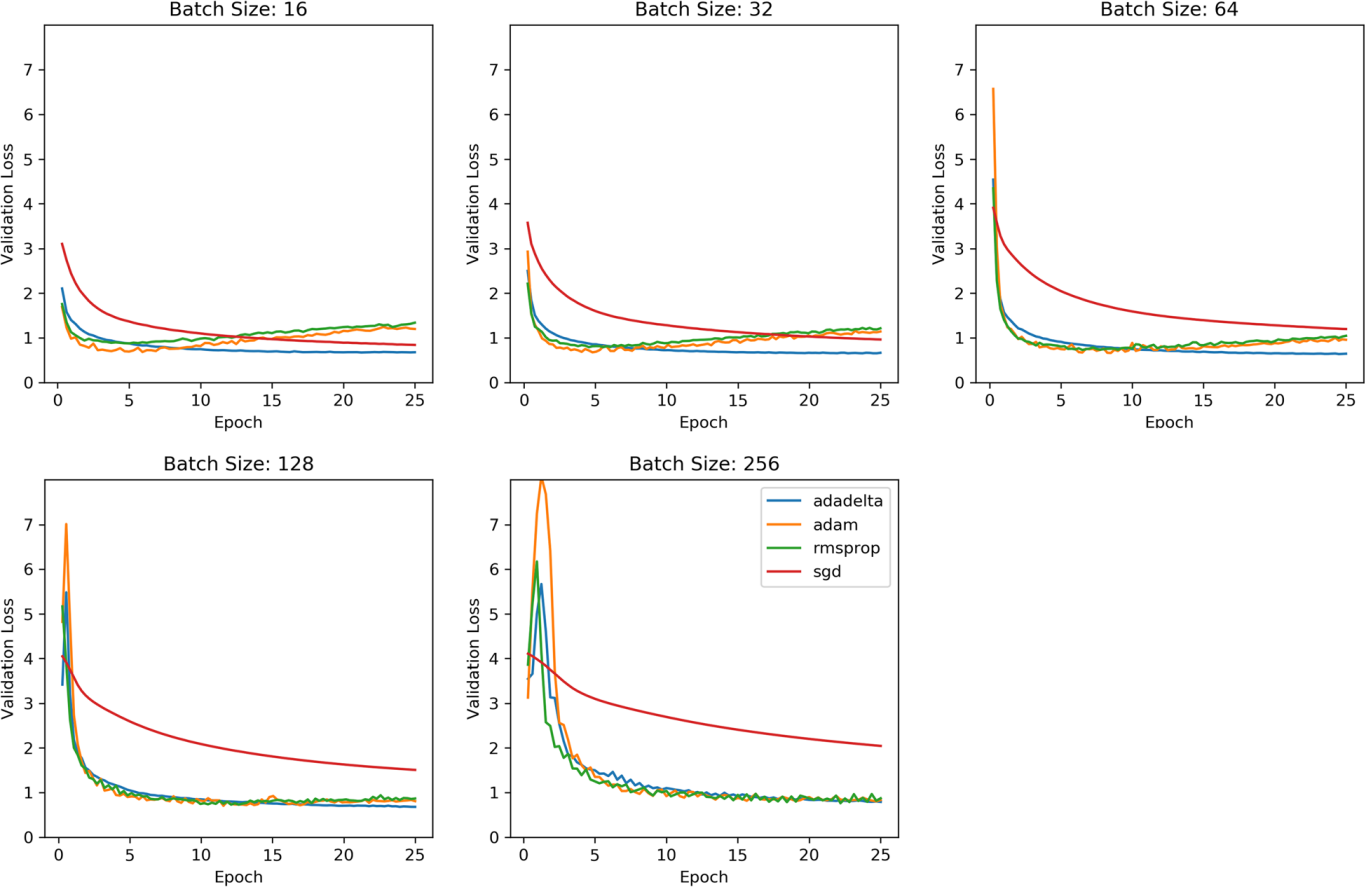
Cross-Entropy Validation Loss vs. Epoch by Mini-batch size

**Fig. 2:**
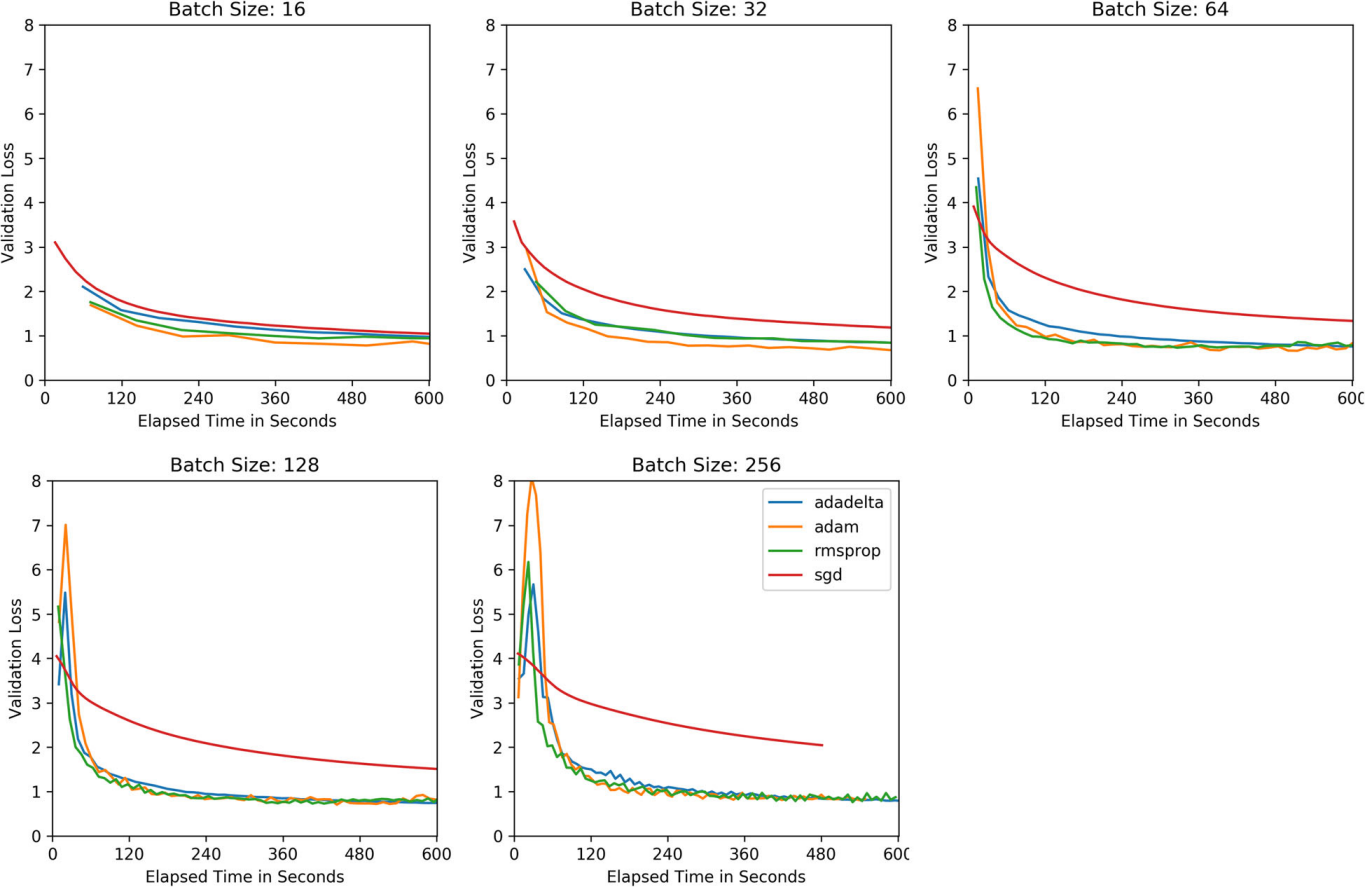
Cross-Entropy Validation Loss vs. Elapsed Train Time by Mini-batch size

**Fig. 3:**
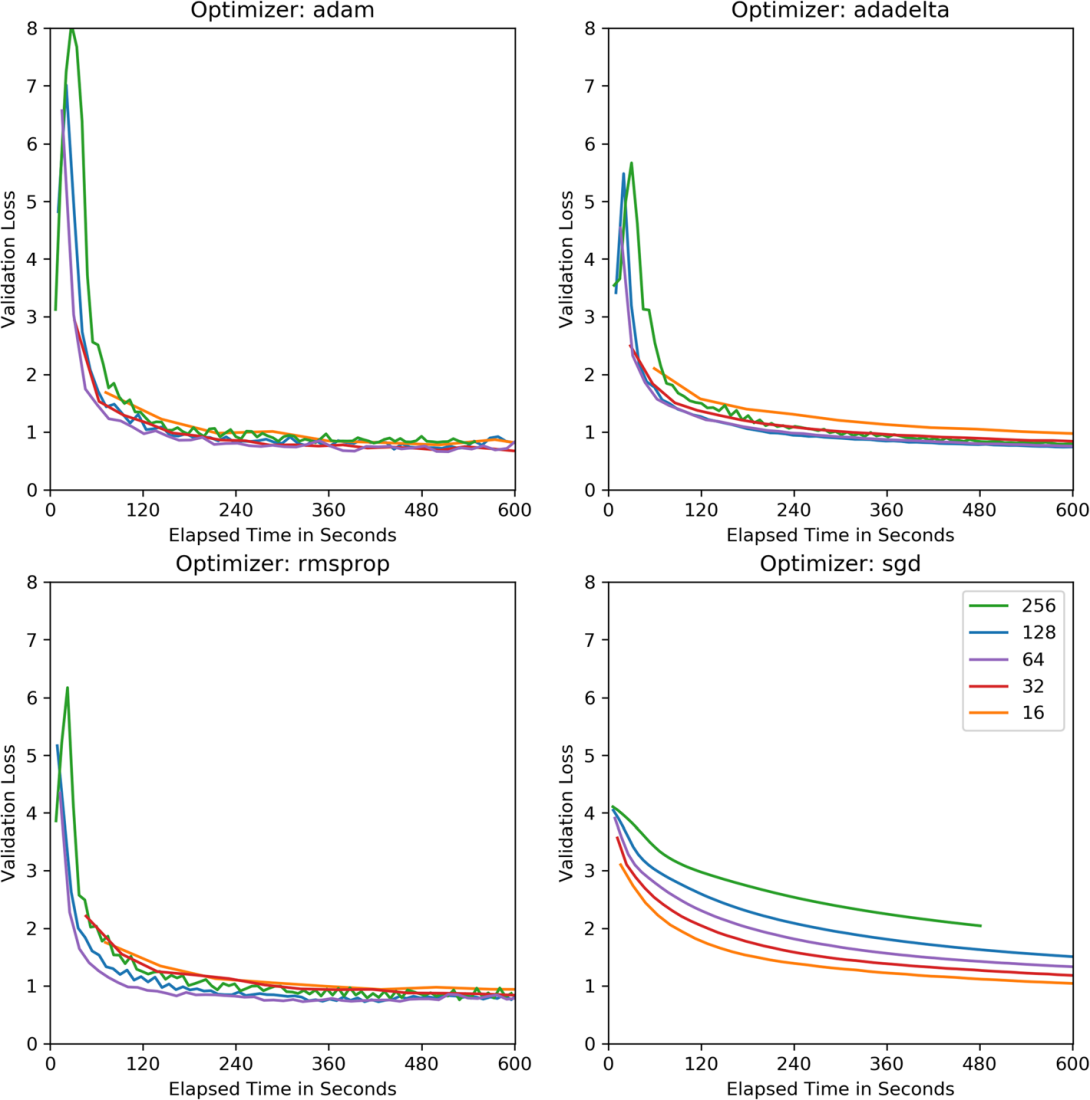
Cross-Entropy Validation Loss vs. Elapsed Train Time by Optimizer Function

Figure 1 plots the validation cross-entropy loss per epoch after 25 epochs of training. This chart compare CNNs trained with each of the four optimizers for a set batch size. Figure 2 compares each optimizer’s validation loss against elapsed training time for each batch size. Figure 3 similarly compares validation loss against elapsed training time, but combines optimizer results to demonstrate more directly how batch size effects training speed for each optimizer.

Across optimizers, the RMSProp optimizer appeared to reach its validation loss minima in the fewest update steps, though this minimum value tended to be consistently higher than Adam and Adadelta’s minimum, with its lowest recorded validation loss of 0.7251 with a batch size of 128. The second fastest converging optimizer was Adam achieving a validation loss of .6670 in training epoch 4.5 using a batch size of 32 but reaching its overall minima validation loss of 0.6579 with a batch size of 64. Ultimately, Adadelta achieved the minimum validation loss for every batch size with the overall minimum validation loss of 0.6337 observed with batch size 64 after 24.25 epochs.

One characteristic of the rapidly converging RMSProp and Adam optimizers is the susceptibility to overfitting, as the validation loss begins to increase without bound. Upon examining the training loss individual on worker nodes, we saw the training loss becoming very close to 0 for each node, providing further evidence of overfitting. Comparing across mini-batch sizes, it appears RMSProp and Adam’s over-fitting trend is much less pronounced for larger training batch sizes. For Adadelta to achieve this typically over twice as many training epochs. A major benefit of Adadelta appears to be its ability to resist overfitting, despite decreasing worker node training loss. One note regarding the basic SGD optimizer is that although it appeared to require many more epochs to come close to matching the other adaptive-rate optimizers, SGD does not require as much time to reach those epochs, likely due to its simple non-parameter specific update rule and its static update rate.

### Scalability

**Fig. 4:**
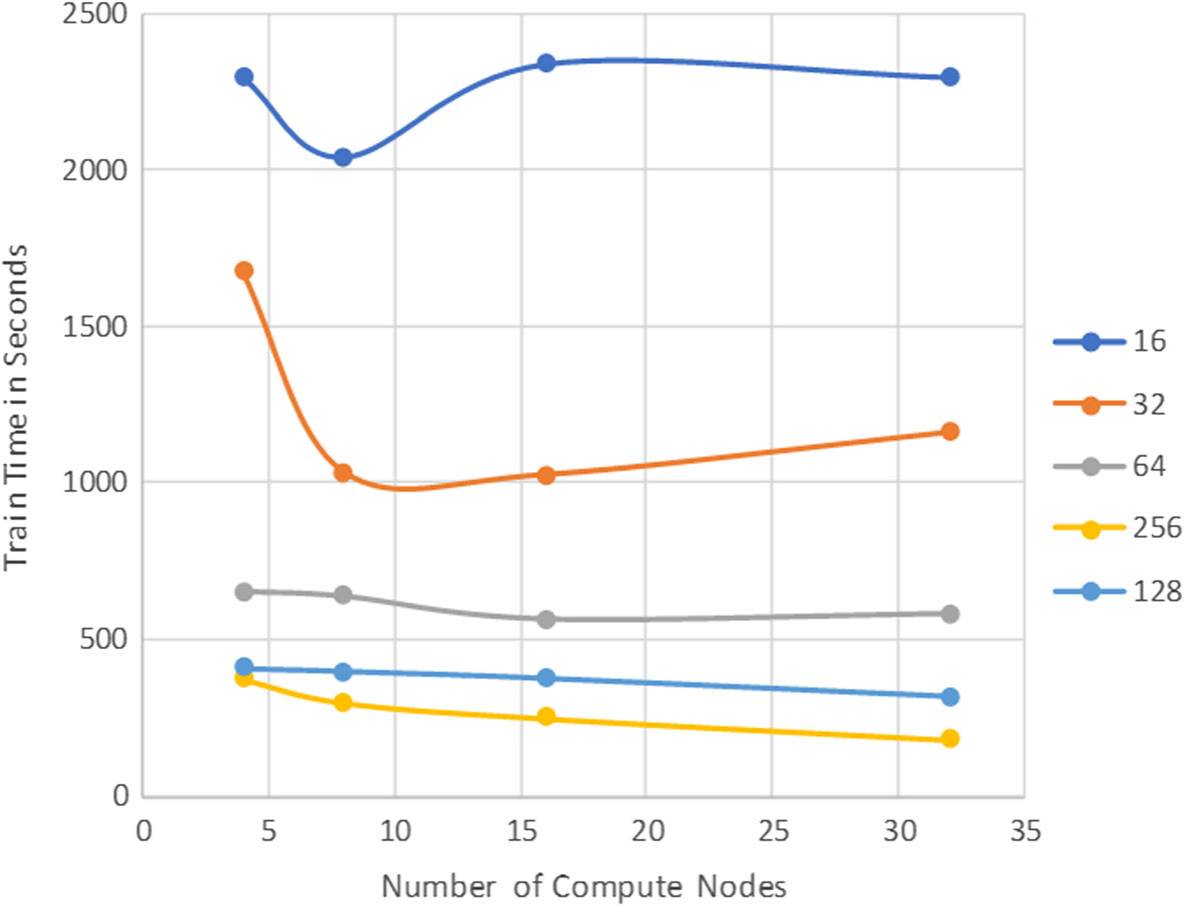
10-Epoch Train Time vs. Number of Worker Nodes for Various Batch Sizes

We compared the training time performance of 4,8,16, and 32 compute nodes by measuring the total elapsed training time for 10 epochs of CNN training and measured the total elapsed training time. To measure the training time impact of additional inter-node communications, we performed each of the above experiments using training batch sizes of 16, 32, 64, 128, and 256, since smaller batch sizes require more frequent inter-node updates. Our results are presented in Fig. 4.

We observed no training time improvements when using more than four compute nodes with smaller mini-batch sizes of 16 or 32. This is due to the inefficiencies resulting from increased inter-node data transfers outweighing the computational performance increases from having multiple nodes. In contrast, when training with larger mini-batch sizes 128 or 256, we observed steady decrease in 10 epoch train times as we added more compute nodes. This result supports the common understanding that the larger mini-batch size generally performs better in implementations of DL training parallelism [8]. These benefits are limited though since larger mini-batch size results in bigger step sizes of the stochastic gradient updates, but the step size cannot exceed the problem-dependent upper bound, which depends on the smoothness of the objective function. Once the mini-batch size has reached this upper bound, increasing the mini-batch size is no longer beneficial to the optimization process.

### Task performance

The CNN classifier consistently outperformed the Random Forest classifier in both micro-averaged and macro-averaged F1 scores (Table 2), demonstrating that the DL-based automatic information extraction is a more effective approach. We observed that the macro-averaged F1 scores are lower than the micro-averaged F1 scores, which is due to the severe class imbalance of the dataset, which inhibits our CNN learning to correctly classify under-represented classes. From the class-specific performance scores listed in Table 3, the largely populated classes such as C50, C61 and C34 had high F1 scores, while under-represented classes such as C74, C12 and C13 were never correctly classified.

**Table 2:** Classification performance of Convolutional Neural Networks and Random Forest classifiers in Micro-F1 and Macro-F1 scores

	Micro-F1	Macro-F1
CNN	0.8425	0.5117
Random Forest	0.7632	0.3567

**Table 3:** Primary site-specific classification performance and their number of support

Site	Precision	Recall	F1-score	Support	Site	Precision	Recall	F1-score	Support
C00	1.00	0.19	0.32	16	C41	0.51	0.24	0.33	88
C01	0.62	0.57	0.59	53	C42	0.92	0.95	0.94	1800
C02	0.71	0.86	0.78	108	C44	0.85	0.91	0.88	1150
C04	0.69	0.64	0.67	39	C48	0.36	0.10	0.15	84
C05	0.62	0.42	0.50	31	C49	0.40	0.44	0.42	261
C06	0.38	0.35	0.37	48	C50	0.94	0.97	0.95	4414
C07	0.83	0.86	0.84	66	C51	0.89	0.74	0.81	97
C08	0.00	0.00	0.00	16	C52	0.43	0.15	0.23	39
C09	0.81	0.90	0.85	71	C53	0.78	0.77	0.77	314
C10	0.36	0.15	0.21	27	C54	0.78	0.91	0.84	882
C11	0.72	0.49	0.58	43	C55	0.47	0.13	0.21	174
C12	0.00	0.00	0.00	11	C56	0.72	0.84	0.77	448
C13	0.00	0.00	0.00	14	C57	0.59	0.16	0.25	83
C14	0.47	0.29	0.36	24	C60	1.00	0.22	0.36	18
C15	0.82	0.81	0.82	199	C61	0.98	0.99	0.98	2313
C16	0.82	0.81	0.81	427	C62	0.98	0.92	0.95	60
C17	0.71	0.54	0.62	156	C64	0.89	0.93	0.91	458
C18	0.85	0.90	0.87	1951	C65	0.33	0.10	0.15	20
C19	0.56	0.31	0.40	118	C66	0.68	0.47	0.56	32
C20	0.81	0.84	0.82	646	C67	0.93	0.96	0.94	947
C21	0.72	0.64	0.68	75	C68	0.33	0.03	0.06	30
C22	0.74	0.82	0.78	268	C69	0.00	0.00	0.00	18
C23	1.00	0.19	0.31	27	C70	1.00	0.06	0.11	17
C24	0.67	0.08	0.14	26	C71	0.79	0.87	0.83	296
C25	0.79	0.79	0.79	151	C72	0.65	0.42	0.51	36
C26	0.00	0.00	0.00	32	C73	0.94	0.97	0.95	305
C30	0.56	0.49	0.52	47	C74	0.00	0.00	0.00	10
C31	0.67	0.13	0.22	15	C75	1.00	0.18	0.31	22
C32	0.79	0.88	0.83	240	C76	0.41	0.27	0.33	196
C34	0.86	0.91	0.88	1569	C77	0.65	0.65	0.65	741
C38	0.62	0.49	0.55	106	C80	0.51	0.48	0.50	962
C40	0.00	0.00	0.00	14	C90	0.00	0.00	0.00	24

**Fig. 5:**
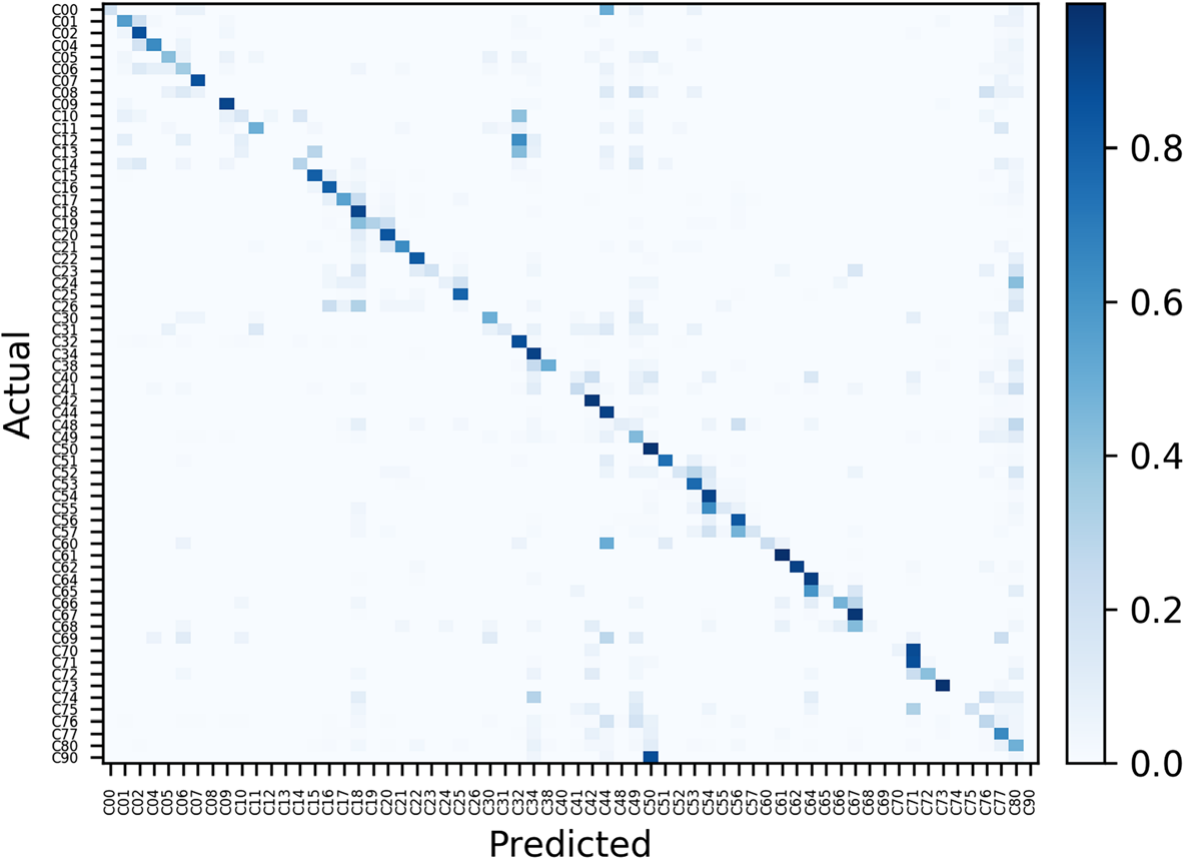
Support-normalized confusion matrix between the actual and predicted values from the CNN classifier for 64 primary cancer sites

We further analyzed these scores using the confusion matrix, illustrated with a normalized color map seen in Fig. 5. For example, we observed that the under-represented classes C12 (Pyriform Sinus) and C13 (Hypopharynx) were misclassified as C32 (Larynx), while the cases in class C70 (Meninges) were misclassified as C71 (Brain). We also noticed that there are relative high false-positives as well as false-negatives for C77 (Lymph nodes) and C88 (Unknown primary site) even with relatively high number of cases.

## Discussion

Mini-batch size is of particular importance because it impacts MPI communication overhead during training as well as the choice of granularity of each update step, serving in an analogous manner to optimizer learn-rate. We demonstrate how training batch size impacts learning scalability since larger batch sizes require fewer parameter updates per epoch, resulting in less messaging overhead time and ultimately reduced training times per epoch. Epoch training time is frequently used in benchmark experiments to represent training performance measured by training data throughput, though this appropriateness can be disputed by comparing the simple SGD optimizer’s fast epoch training times against its poor real-time validation loss. We also reveal limitations of the more sophisticated update rules, the often initially poor performing and for larger batch sizes, detrimental updates learned in initial epochs as these optimizers appropriately adjust their adaptive learn rate. Conversely, because smaller batch sizes result in more fine - grained parameter read and write updates, every optimizer converges in substantially fewer updates but requiring disproportionally larger amounts of time.

**Fig. 6:**
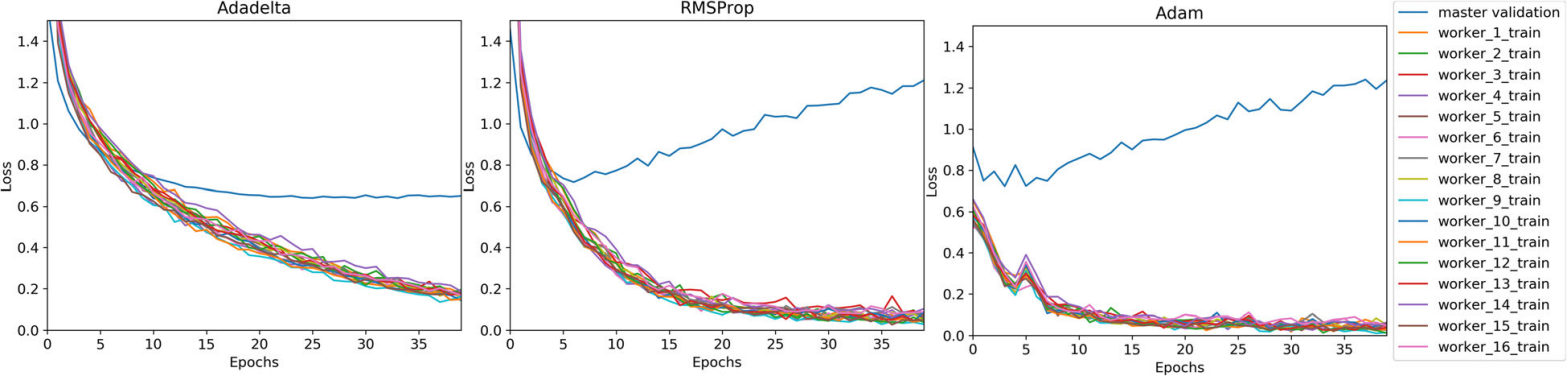
Master Node Validation Loss/Worker Node Training Loss vs Epoch with batch size 64

Other key observations include the remarkable divergence of the RMSProp and Adam optimizers. The Adam optimizer is especially popular in current deep learning literature, but pragmatic researchers may tolerate training instability from Adam’s rapid convergence. In our experiments, we found that though the Adadelta optimizer achieved the lowest overall validation loss in our experiments, and that the Adam optimizer would reach a competitive second lowest validation loss but required less than one third of the training updates Adadelta required. From our experimental data, we can explain these learning patterns as resulting from how each optimizer’s learning rate adaptation affects overfitting. Figure 6 compares worker node training batch training loss against the parameter server’s tested validation loss. Both RMSProp and Adam use constantly diminishing learn rates by dividing the initial rate by the exponentially decaying average of squared gradients. Adam additionally multiplies the learn rate with the exponentially decaying average of squared gradients, resulting in a more responsive learn rate adaptation, faster training convergence and more extreme overfitting. Comparatively, Adadelta’s learn rate is scaled by a simple running average of gradients retained within a fixed number of updates. This less responsive mechanism explains why Adadelta converges significantly slower than RMSProp and Adam but is highly resistant to overfitting.

An issue relevant to our study and the bioinformatics domain is extreme class imbalance, as our most prevalent class, breast cancer, had 4415 (C50) cases, while the least prevalent classes, adrenal gland cancer (C74) and pyriform sinus cancer (C12), had only 10 cases and 11 cases respectively. Though some worker nodes may never directly interact with minority classes, they would still indirectly learn from these cases via parameter server updates. A potential future solution to class imbalance is oversampling the minority classes so that each worker node may have some cases of the class, with the many worker nodes helping to reduce the downside of longer training times as a result of additional training cases, though care must be made to ensure balanced worker node train times for any asynchronous implementation.

Because OLCF’s Titan architecture consists of a single GPU per compute node, we utilized a simple worker hierarchy consisting of a single master node which all worker nodes submit updates to. Future compute clusters such as OLCF Summit have configurations of 4 GPU devices on each node, each capable of accessing the node’s shared memory. This difference in inter-device bandwidth warrants future investigation to identify optimal worker-master configurations across heterogeneous architectures.

In this paper, we applied an implementation of data parallelism to CNN training and demonstrated the scalability of this method to our cancer surveillance task. Such techniques are particularly useful to the surveillance community, but they require many training epochs with vast amounts of training samples which may be too large for a single node computer or a multiple-GPU workstation. We examined the scalability of the data parallelism using the Titan supercomputer and evaluated task performance on the information extraction of primary cancer sites from the cancer pathology reports that are free form natural language texts. Our suggested method outperformed a traditional machine learning algorithm, and scales to multiple nodes by communicating via the MPI protocol.

The study leads us into further investigations and analyses such as training efficiency of DL networks, actual throughput of the communication between compute nodes, efficient load balancing between compute nodes, and even the proper choice of DL libraries apt to implement data parallelism. Along with the hyperparameter optimization efforts and the data parallelism of CNN for large scale natural language processing, the HPC environment is critically important for supporting NLP applications for the biomedical and other application domains.

## Conclusions

To scale deep learning methods to very large data sets, we have implemented a data parallel approach for network training within an HPC environment. We used the convolutional neural network for natural language processing to extract ICD-O-3 from a large-scale cancer pathology reports as our benchmarking task.

We compared how training parameters, including optimizer and batch size, impact training performance which was measured in elapsed time and number of training epochs required to minimize validation loss. The Adadelta optimizer achieved the lowest validation loss of 0.6337 with 16 worker nodes and a training batch size of 64 after over 24 training epochs. The Adam optimizer achieved a minimum loss of .6670 but converged significantly faster, in only 4.5 training epochs with a batch size of 32.

We measured training scalability by comparing how worker node count affected total training time. We found that training batch size strongly affected scalability, with smaller batch sizes of 16 and 32 resulting in reduced training times using up to 8 nodes and additional workers causing longer train times. With a batch size of 64, we improved scalability and reduced training times with up to 16 nodes. Our largest tested batch sizes of 128 and 256 improved scalability up to 32 nodes.

Finally, we demonstrated the effectiveness of our CNNs against a more conventional statistical learning model in a 10-fold cross validated experiment. We used a Random Forest classifier with a TF-IDF document representation and found the CNN trained with data-parallelism outperformed the Random Forest model resulting in micro-averaged F1 scores of .8425 and .7632 and macro-averaged F1 scores of .5117 and .3567 respectively.

Our results suggest that the scalability of our deep learning model to multiple computational nodes is possible but subject to training parameters. We found the major bottleneck for scalability is worker-parameter node communication, since larger batch sizes require fewer updates per epoch. We also see a tradeoff between scalability and training performance, since the larger, more scalable batch sizes require more epochs to converge to less-optimal losses. Future research should attempt to improve the scalability-learning performance tradeoff. Since increased inter-node messaging appears to be major limitation to training scalability, using a modified update scheme such as using multiple parameter servers or reducing worker update message size with a model parallelism implementation.

## Abbreviations

AdaDelta: Modified Adaptive Gradient
Adam: Adaptive Moment Estimation
CNN: Convolutional Neural Network
DL: Deep Learning
DNN: Deep Neural Network
GPU: Graphics Processing Unit
HPC: High Performance Computing
ICD-O-3: International Classification of Diseases for Oncology, Third Edition
MPI: Message Passing Interface
NAACCR: North American Association of Central Cancer Registries
NLP: Natural Language Processing
OLCF: Oak Ridge Leadership Computing Facility
RMSProp: Root Mean Square Propagation
SEER: Surveillance, Epidemiology, and End Results Program
SGD: Stochastic Gradient Descent
TF/IDF: Term Frequency / Inverse Document Frequency

## References

[1] Min S, Lee B, Yoon S (2017). Deep learning in bioinformatics. Brief Bioinform.

[2] LeCun Y, Bengio Y, Hinton G (2015). Deep learning. Nature.

[3] Le QV, Ngiam J, Coates A, Lahiri A, Prochnow B, Ng AY (2011). On optimization methods for deep learning. Proceedings of the 28th International Conference on Machine Learning.

[4] Smith TJ, Davidson NE, Schapira DV, Grunfeld E, Muss HB, Vogel VG (1999). American Society of Clinical Oncology 1998 update of recommended breast cancer surveillance guidelines. J Clin Oncol.

[5] Siegel RL, Miller KD, Jemal A (2016). Cancer statistics, 2016. CA Cancer J Clin.

[6] Chu CT, Kim SK, Lin YA, Yu Y, Bradski G, Olukotun K (2007). Map-reduce for machine learning on multicore.

[7] Tsitsiklis J, Bertsekas D, Athans M (1986). Distributed asynchronous deterministic and stochastic gradient optimization algorithms. IEEE Trans Autom Control.

[8] Dean J, Corrado G, Monga R, Chen K, Devin M, Mao M (2012). Large scale distributed deep networks. In: advances in neural information processing systems.

[9] Tieleman T, Hinton G (2012). Lecture 6.5-rmsprop: divide the gradient by a running average of its recent magnitude. COURSERA: Neural networks for machine learning.

[10] Zeiler MD (2012). ADADELTA: an adaptive learning rate method. arXiv preprint arXiv:12125701.

[11] Kingma DP, Ba J (2014). Adam: A method for stochastic optimization. arXiv preprint arXiv:14126980.

[12] Penberthy LT, Winn DM, Scott SM. Cancer surveillance informatics. In: Oncology Informatics. Boston: Academic Press; 2016. p. 277–285.13.

[13] Carrell DS, Halgrim S, Tran DT, Buist DS, Chubak J, Chapman WW (2014). Using natural language processing to improve efficiency of manualchart abstraction in research: the case of breast cancer recurrence. Am J Epidemiol.

[14] Li Y, Martinez D (2010). Information extraction of multiple categories from pathology reports. Proceedings of the Australasian Language Technology Association Workshop.

[15] Salton G, Wong A, Yang CS (1975). A vector space model for automatic indexing. ACM computing surveys (CSUR).

[16] Mikolov T, Sutskever I, Chen K, Corrado GS, Dean J (2013). Distributed representations of words and phrases and their compositionality.

[17] Kim Y (2014). Convolutional neural networks for sentence classification. arXiv preprint arXiv:14085882.

[18] Tarditi D, Puri S, Oglesby J (2006). Accelerator: using data parallelism to program GPUs for general-purpose uses. ACM SIGPLAN Not.

[19] Fritz AG. In: International classification of diseases for oncology: ICD-O, vol. 20. Geneva: World Health Organization Press; 2000.

[20] Zhang Y, Wallace B (2015). A sensitivity analysis of (and practitioners’ guide to) convolutional neural networks for sentence classification. arXiv preprintarXiv:151003820.

[21] Qiu JX, Yoon HJ, Fearn PA, Tourassi GD (2018). Deep learning for automated extraction of primary sites from cancer pathology reports. IEEE journal of biomedical and health informatics.

[22] Bottou L. Large-scale machine learning with stochastic gradient descent. In: Proceedings of COMPSTAT’2010. Heidelberg: Physica-Verlag; 2010. p. 177–186.23.

[23] Anderson D, Vlimant JR, Spiropulu M. An MPI-Based Python Framework for Distributed Training with Keras. arXiv preprint arXiv:171205878.2017;.24.

[24] Chollet F (2013). Keras. GitHub.

[25] Abadi M, Barham P, Chen J, Chen Z, Davis A, Dean J (2016). TensorFlow: A System for Large-Scale Machine Learning. Operating Systems Design and Implementation.

[26] Piskorski J, Yangarber R. Information extraction: Past, present and future. In: Multi-source, multilingual information extraction and summarization. Berlin: Springer; 2013. p. 23–49.

